# Novel Multiparametric Magnetic Resonance Imaging-Based Deep Learning and Clinical Parameter Integration for the Prediction of Long-Term Biochemical Recurrence-Free Survival in Prostate Cancer after Radical Prostatectomy

**DOI:** 10.3390/cancers15133416

**Published:** 2023-06-29

**Authors:** Hye Won Lee, Eunjin Kim, Inye Na, Chan Kyo Kim, Seong Il Seo, Hyunjin Park

**Affiliations:** 1 Samsung Medical Center, Department of Urology, Sungkyunkwan University School of Medicine, Seoul 06351, Republic of Korea; hyewon3839.lee@samsung.com; 2 Department of Electrical and Computer Engineering, Sungkyunkwan University, Suwon 16419, Republic of Korea; 3 Department of Radiology and Center for Imaging Science, Samsung Medical Center, Sungkyunkwan University School of Medicine, Seoul 06351, Republic of Korea; chankyokim@skku.edu; 4 Center for Neuroscience Imaging Research, Institute for Basic Science, Suwon 16419, Republic of Korea

**Keywords:** prostate cancer, radical prostatectomy, biochemical recurrence, survival prediction, magnetic resonance imaging, radiomics, deep learning

## Abstract

**Simple Summary:**

Existing research on predicting biochemical recurrence after prostate surgery has been insufficient. Here, we aimed to predict biochemical recurrence after radical prostatectomy leveraging recent advances in deep learning. We combined clinical variables with multiparametric magnetic resonance imaging using deep learning methods. Our method performed better than existing methods. Our method could direct patients to individualized care using routine medical imaging and could achieve better patient care.

**Abstract:**

Radical prostatectomy (RP) is the main treatment of prostate cancer (PCa). Biochemical recurrence (BCR) following RP remains the first sign of aggressive disease; hence, better assessment of potential long-term post-RP BCR-free survival is crucial. Our study aimed to evaluate a combined clinical-deep learning (DL) model using multiparametric magnetic resonance imaging (mpMRI) for predicting long-term post-RP BCR-free survival in PCa. A total of 437 patients with PCa who underwent mpMRI followed by RP between 2008 and 2009 were enrolled; radiomics features were extracted from T2-weighted imaging, apparent diffusion coefficient maps, and contrast-enhanced sequences by manually delineating the index tumors. Deep features from the same set of imaging were extracted using a deep neural network based on pretrained EfficentNet-B0. Here, we present a clinical model (six clinical variables), radiomics model, DL model (DLM-Deep feature), combined clinical–radiomics model (CRM-Multi), and combined clinical–DL model (CDLM-Deep feature) that were built using Cox models regularized with the least absolute shrinkage and selection operator. We compared their prognostic performances using stratified fivefold cross-validation. In a median follow-up of 61 months, 110/437 patients experienced BCR. CDLM-Deep feature achieved the best performance (hazard ratio [HR] = 7.72), followed by DLM-Deep feature (HR = 4.37) or RM-Multi (HR = 2.67). CRM-Multi performed moderately. Our results confirm the superior performance of our mpMRI-derived DL algorithm over conventional radiomics.

## 1. Introduction

Radical prostatectomy (RP), the first-line treatment for organ-confined or locally advanced prostate cancer (PCa), yields excellent long-term survival outcomes [1,2,3]. Post-RP biochemical recurrence (BCR), affecting 50% of the patients, is a strong surrogate marker for indicating subsequent distant metastasis, as well as PCa-specific and overall mortality [1,4]. BCR predominantly develops in patients with high-risk features, such as high initial prostate-specific antigen (PSA) levels, adverse RP pathology, including extracapsular extension (ECE) and seminal vesicle invasion (SVI), positive surgical margins (PSM), and surgical Gleason score (GS) ≥8 [1,4,5,6]. However, the natural history of relapse following RP is heterogeneous even in patients with high-risk features, reflecting high heterogeneity in clinical, tumor pathophysiological, and molecular aspects of PCa [4,7]. Therefore, early identification of patients with PCa at a higher risk for developing post-RP BCR is important for intensive monitoring or providing adjuvant therapies at the proper time for the control of systemic micro-metastasis for achieving long-term survival benefits.

Generally, multiparametric magnetic resonance imaging (mpMRI) integrates conventional anatomic sequences (T1- and T2-weighted imaging) with functional sequences, such as diffusion-weighted MRI (DWI), including the calculation of apparent diffusion coefficient (ADC) and dynamic contrast-enhanced (DCE) maps, and optionally, MR spectroscopy [8,9,10,11,12]. Owing to its superior spatial resolution, mpMRI is regarded as the most sensitive and specific imaging method for clinical staging, preoperative risk stratification, surgical planning, predicting the presence of clinically significant PCa, and monitoring recurrence following RP [1,3,4,7,8,9,10,11,12,13,14,15,16]. Noninvasive radiological assessment of anatomical, functional, and physiologic information about the entire prostate through preoperative mpMRI can overcome the known molecular heterogeneity and multifocality of PCa that can be neglected by conventional tools incorporating only clinicopathological factors [7,8,9,10,11,12].

The extraction of handcrafted radiomics features based on labeled PCa regions of interest (ROIs) on mpMRI allow objective qualitative and quantitative characterization of tumor phenotypes [7,10,15,16,17,18,19,20]. PCa radiomics using preoperative mpMRI has a high potential to define tumor aggressiveness and facilitate better prognostication of PCa-specific outcomes, including BCR and post-RP BFS [7,10,15,17,18,19,20,21]. However, the radiomics approach using handcrafted radiomics features typically considers only the features of an index lesion although PCa is a multifocal disease [7,10,15,17,18,19]. Additionally, the challenges of a typical radiomics workflow, such as defining and contouring ROI, selecting the best extraction features, and appropriate predictive modeling still remain [7,10,15,16,20].

Recently, deep learning (DL) models, which have yielded excellent results for the analysis of mpMRI frequently outperform radiomics studies [22,23,24,25,26,27,28,29,30]. This is because DL models condense high-dimensional imaging data into few representative features, creating classifier models via data mining with more powerful fitting capabilities, based predominantly on convolutional neural networks (CNNs) directly applied to real images [22,23,24,25,26,27,29,30,31,32,33]. DL models typically do not require precise ROI annotation or prior hand-crafted radiomics feature extraction as the algorithm automatically learns to extract complex and abstract features beyond radiomics features during training, thus reducing human effort and interobserver variability, compared to manual ROI annotation [22,23,24,25,30]. Furthermore, DL models are often based on entire organ-specific images, which contain information on not only microstructural anatomical information but also information representing the underlying pathophysiologic process such as the relationship of the tumor and the tumor microenvironment (TME) [22,23,24,25,26,27,28,29,32,33]. Recently, DL models using prostate mpMRI have shown promising results in detecting clinically significant cancers and predicting PCa aggressiveness and oncologic prognosis [22,23,25,26,27,30,33,34,35,36].

Accurate prediction of post-RP BCR requires long-term follow-up (>10 years) owing to the high variability of BCR events and PCa’s slow-growing and often indolent nature [37,38]. More importantly, the characteristics of PCa exhibit regional and ethnic variations, suggesting that race- and nation-specific characteristics of PCa should be considered for developing a more accurate prediction model. Asian men, especially Koreans, are more likely to possess unfavorable risk profiles, such as advanced pathological stage, high GS, high PSA levels, and worse prognoses than Americans and Europeans [39,40,41,42]. The performance of conventional clinical predictive tools in the Asian population, including Koreans, has been reported as suboptimal compared to those of the European and American populations [34,43,44,45]. These limitations motivated us to develop a new system for predicting long-term post-RP BFS in Korean patients with high-risk PCa according to our own data by completely integrating all the available clinical variables and mpMRI features that could be clinically useful. To date, researchers have not developed clinically relevant mpMRI-based DL risk models for predicting post-RP long-term BCR-free survival because the erstwhile standard protocol of PCa care did not involve preoperative mpMRI before RP in most countries. Notably, in contrast to other hospitals, preoperative prostate mpMRI has routinely been performed before RP in patients with localized PCa at our institution since 2008 [46], enabling the accumulation of large-scale, high-quality mpMRI datasets in addition to long-term follow-up clinical data for developing more dependable mpMRI-based DL risk models.

## 2. Materials and Methods

### 2.1. Patient Selection

This monocentric study was approved by the Institutional Review Board of Samsung Medical Center (SMC) (no. 2022-01-134), which waived the requirement for written informed consent owing to the retrospective study design. The study protocols were conducted in accordance with the tenets of the Declaration of Helsinki. The inclusion criteria were as follows: (1) primary prostate acinar adenocarcinoma confirmed by RP; (2) undergoing RP after 3 T prostate mpMRI; (3) mpMRI images of adequate quality available in the Picture Archiving Communication System (Centricity; GE Healthcare, Chicago, IL, USA); (4) no history of neoadjuvant or adjuvant therapies for PCa; (5) available complete pathological and follow-up information. The exclusion criteria were as follows: (1) incomplete mpMRI or poor-quality MRI scans; (2) PSA persistence characterized by a postoperative PSA nadir >0.04 ng/mL 3 months post RP; (3) missing clinical variables; (4) tumors of other pathological types or mixed pathology. The primary endpoint was the prediction of post-RP BCR-free survival. Post-RP BCR was defined as an initial PSA value ≥ 0.2 ng/mL, confirmed by a subsequent PSA value of ≥0.2 ng/mL [1,3,4,5,47]. The time of BCR incidence was defined as the midpoint between the nadir and the first of three consecutive elevations [47]. BCR-free survival was defined as the interval between the date of RP and that of BCR [47]. We censored the data of patients who were still alive but without BCR at the last reported follow-up.

**Figure 1:**
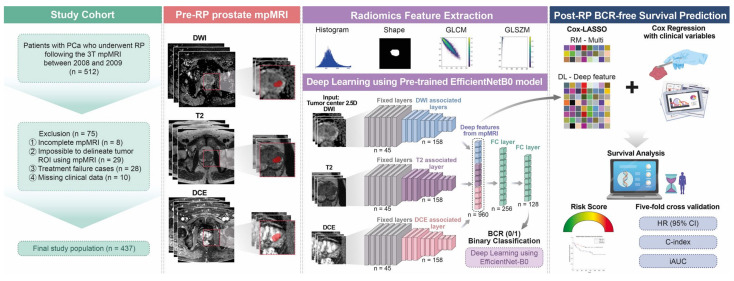
Overall framework of this study for data preparation, modeling, and predicting BCR-free survival. BCR = biochemical recurrence; RP = radical prostatectomy; PCa = prostate cancer; T = Tesla; mpMRI = multiparametric magnetic resonance imaging; ROI = region of interest.

Initially, we retrospectively reviewed the baseline clinicopathological data of 512 patients with pathologically confirmed PCa who underwent RP, with or without lymphadenectomy, at the SMC between 2008 and 2009. RP was performed by five surgeons with varying surgical experience at our institution. The decision to perform lymphadenectomy was based on the preoperative lymph node involvement risk assessment and surgeon’s discretion. Participants were followed up every 3 months during the first 2 years, every 6 months between the second and fifth years, and annually thereafter. Finally, a total of 437 patients were analyzed. The study flowchart is depicted in Figure 1.

### 2.2. mpMRI Acquisition Protocol and Interpretation

All the patients underwent prostate mpMRI using a 3 T MRI scanner (Achieva TX; Philips Healthcare, Best, The Netherlands) equipped with a phased-array coil. Routine mpMRI protocols included T1WI, T2WI, DWI, ADC maps, and DCE sequences, acquired following intravenous injection of gadolinium diethylenetriamine penta-acetic acid (Gadovist; Schering, Berlin, Germany) [46,48]. Table 1 summarizes additional details regarding image acquisition. All the mpMRI scans were reviewed by an experienced uroradiologist (C.K.K.) with 14 years of experience in prostate MRI interpretation.

### 2.3. Clinical Variable-Based Risk Model Construction

Figure 1 shows the study’s conceptual workflow. First, we built a clinical variable risk model (denoted as CM) comprising six clinically important variables for post-RP BCR, namely, the patients’ age during RP, preoperative PSA level, ECE, SVI, PSM, and surgical GS International Society of Urological Pathology (ISUP) grade, to predict the BCR-free survival using a Cox proportional hazards model. We assigned surgical GS values to the corresponding ISUP grade to create an ordinal classifier ranging from 1 to 5 instead of the grade group as follows: grade 1 = GS 3 + 3, grade 2 = GS 3 + 4, grade 3 = GS 4 + 3, grade 4 = GS 8 (4 + 4, 3 + 5, or 5 + 3), and grade 5 = GS 9 (4 + 5, 5 + 4) or GS 10 [49].

### 2.4. Tumor ROI Delineation, Radiomics Feature Extraction, Selection, and Risk Model Generation

**Figure 2:**
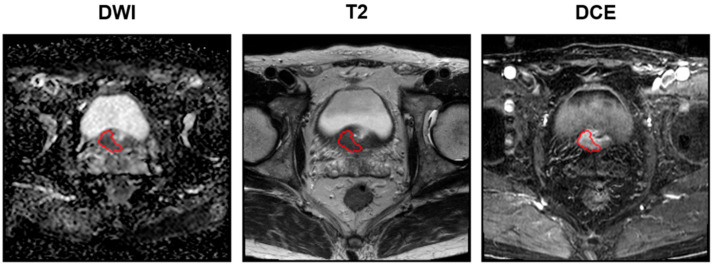
Examples of delineation on mpMRIs: DWI (**left**), T2 (**middle**), and DCE (**right**) sequences. The red circles denote the tumor ROIs. Images acquired on a Philips 3T Scanner. Scan magnification scale: ×1. The patient’s information: post-RP BCR (+), initial PSA of 13.32 mg/dL, mpMRI: suspicious ECE (cT3a), surgical pathology findings: GS of 9 (4 + 5), ECE (pT3a), no lymph node involvement (pN0), PSM. mpMRI = multiparametric magnetic resonance imaging; DWI = diffusion-weighted image; T = Tesla; DCE = dynamic contrast-enhanced; RP = radical prostatectomy; BCR = biochemical recurrence; PCa = prostate cancer; GS = Gleason score; ECE = extracapsular extension; PSM = positive surgical margin.

For each patient, tumor delineation was performed by manually placing the ROI on T2WI, ADC map, and DCE images, according to the histopathological results (Figure 2). Tumor ROI was depicted for each patient’s index lesion using a three-dimensional (3-D) slicer. The lesion with the largest diameter or that demonstrating ECE was considered the index lesion. Tumor ROI measurement encompassed the maximum possible lesion extent in the image with the greatest visibility.

We extracted radiomics features from the ROIs drawn on the T2WI, ADC, and DCE sequences of each patient using the open-source software, Python package “Pyradiomics” (version 3.0.1) [50]. Seventy-two radiomics features were extracted and divided into three categories: (1) 14 shape-based features, including the ROI’s 3D size and shape descriptors; (2) 18 first-order features describing the voxel intensity distribution within the ROI; (3) 40 textural features, including 24 gray-level co-occurrence matrix and 16 gray-level size zone matrix features (Figure 1). The radiomics features from the test set of each fold were z-score-normalized by applying the mean and standard deviation values of the training set. Details of the training and test set splits are described in Section 2.6. We constructed a radiomics risk model based on the radiomics features extracted from mpMRI for BCR-free survival prediction (RM-Multi) (Figure 1). We adopted a Cox model regularized with the least absolute shrinkage and selection operator (Cox-LASSO) logistic regression analysis using fivefold cross-validation to select an optimized subset of radiomics features with nonzero coefficients and reduce overfitting and selection bias while constructing the final model (Figure 1 and Figure S1). We also constructed a combined risk model by adding six clinical variables to the RM-Multi model using a similar Cox-LASSO approach to assess the impact of the clinical parameters (CRM-Multi) (Figure 1).

### 2.5. DL Procedures and Construction of the Related Risk Models

**Figure 3:**
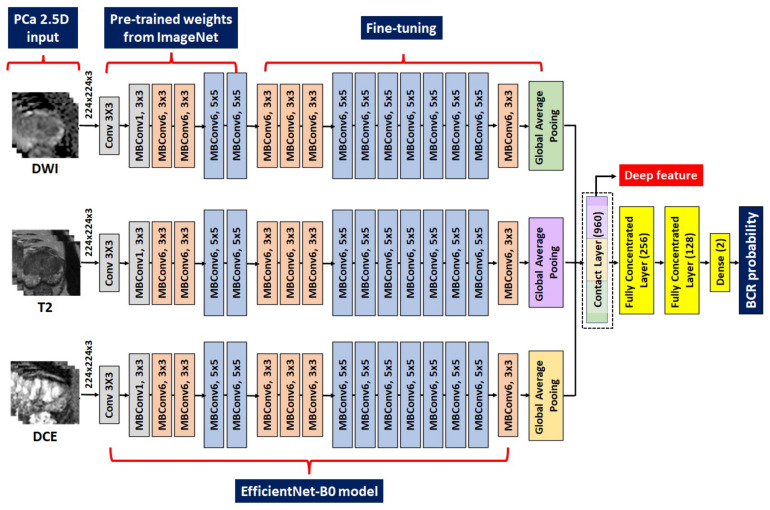
Details of the adopted EfficieneNetB0 model. The left figure denotes the multimodal MRI input of three slices. The second section from the left is the frozen layers pretrained from the ImageNet. The third section from the left is the fine-tuned layers with our local data. The right figure denotes the procedures to combine deep features and classify the binary BCR status. DWI = diffusion-weighted image; T = Tesla; DCE = dynamic contrast-enhanced; MRI = magnetic resonance imaging; BCR = biochemical recurrence.

In this novel study, we developed a deep neural network for predicting post-RP BCR-free survival following the process outlined in Figure 1 and Figure 3. First, we extracted deep features by cropping the raw mpMRI images of T2WI, DWI with ADC, and DCE sequences of each patient into a dataset of 2.5D images as input for the DL model. The axial slice depicting the largest tumor and two additional axial slices above and below the center slice were used to sample the tumor characteristics with varying z-dimensions for each MRI modality. We normalized the intensity for each slice to 0–1. We extracted an image patch of 64 × 64 pixels centered on the tumor centroid from the center slice, since a 64 × 64 patch size was determined to contain the largest tumor. Two additional 64 × 64 patches were extracted from the upper and lower slices. The regular neural network of the natural images procured a three-channel (i.e., red, green, and blue channels) input as default. Our input consisted of three stacked axial patches, which were matched to the three-channel input of the natural image (Figure 1 and Figure 3).

Thereafter, we constructed a neural network to predict the binary BCR status, not BCR-free survival. The CNN architecture was characterized by connected nonlinear functions that learn multiple levels of representations of the input data, thereby yielding millions of possible features using a raw image [23,25,27,29,31,32,51]. In general, the performance of the DL models relies on the vast number of sample images because a large dataset allows improvement of generalizability and performance by training deeper networks [29,31,32,51]. In contrast, a key limitation is the massive number of sample images required to robustly train a model. EfficientNet, one of the most potent CNN architectures, uses a compound scaling method to increase the network depth, width, and resolution, while requiring fewer computational resources than other models [52,53]. Hence, we used an EfficientNet-B0 network pretrained from the vast ImageNet natural image database as a backbone network to handle the raw mpMRI input and partially mitigate the sample size issue of DL. The DL deep features of the intermediate layer were used to build a risk model (Figure 1 and Figure 3). The first 45 layers were frozen, and the subsequent 158 layers were fine-tuned using local data, resulting in 320 features for each modality (Figure 1 and Figure 3). The amalgamation of the three modalities yielded 960 deep features, which were used to predict the binary BCR status from two fully connected layers (Figure 1 and Figure 3). Second, the deep features from the intermediate layer contained useful information for predicting BCR, which could be beneficial for BCR-free survival analysis. Consequently, these deep features were subjected to Cox-LASSO, yielding a DL deep feature risk model (DLM-Deep feature). Collating DL’s deep features with six clinical variables resulted in a combined DL risk model (CDLM-deep feature) using the previously described approach. Furthermore, we applied gradient-weighted class activation mapping (Grad-CAM) to interpret the DL model for BCR prediction, which was consequently applied to the last convolutional layer.

### 2.6. Statistical Analysis

We performed Student’s *t*-test and the chi-square or Fisher’s exact test for the numerical and categorical variables, respectively, to compare the clinical characteristics of the patients with and without BCR. Quantitative variables are presented as the median (interquartile range [IQR]) or mean (standard deviation [SD]), whereas qualitative variables are presented as absolute values (percentages). We performed a univariate analysis to confirm the relevance of each clinical variable for BCR. Furthermore, Kaplan–Meier (K–M) curves were utilized to compare the ability of the risk models to assign a high and low risk of 10-year BCR-free survival using the median risk score as threshold. We used the hazard ratio (HR) with 95% confidence interval (CI) and log-rank tests to compare the two risk groups. The C-index measured the rank correlation between the risk score and BCR-free survival. The C-index value was higher if the time to BCR was shorter in patients at a higher risk than in patients at lower risk. Lastly, we calculated the integrated time-dependent area under the curve (iAUC), a time-wise AUC average for predicting events.

We split our data into two groups, the training and test sets, in a fivefold cross-validation manner. Thus, our data were split into five parts, where four parts were used for training, while the remaining part was used for testing. By repeating the procedure for the five different test sets and retaining the same ratio of BCR to non-BCR cases (1:3) across all test sets, potential overfitting caused by the relatively small cohort was reduced (Table S1). We computed the average and SD of HRs with 95% CIs, C-indices, and iAUCs across all repetitions to obtain the final scores. The performance of each risk model for predicting post-RP BCR-free survival was compared to the average HRs (95% CI), C-indices, and iAUCs in a fivefold cross-validation. Two-sided *p*-values <0.05 were considered statistically significant. All statistical analyses were performed using Python (version 3.6.8; Python Software Foundation, Wilmington, DE, USA).

## 3. Results

Table 2 summarizes the major clinicopathological characteristics of all the patients (median age, 66 years; mean preoperative PSA level, 7.61 mg/dL). BCR occurred in 110 (25.2%) of the 437 patients following a median BCR-free survival period of 23 months (IQR: 9–52 months) during a median follow-up period of 61 months (IQR: 24–110 months). Significant differences in the clinicopathological risk factors associated with BCR (preoperative PSA, ECE, SVI, SM, and pathologic GS ISUP grade; all *p* < 0.001) were observed between the BCR and non-BCR groups, which was consistent with previous research [1,2,3,4,6,54].

**Figure 4:**
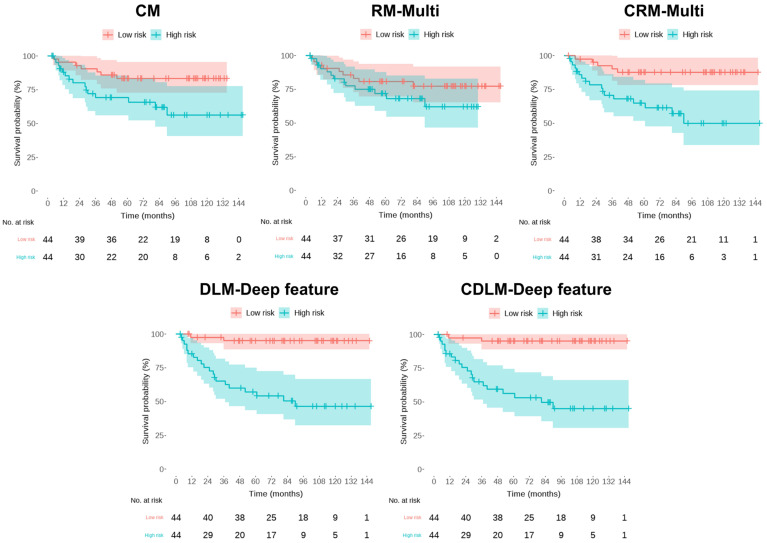
Kaplan–Meier’s estimates of biochemical recurrence -free survival using the five risk models. Each plot has a *p*-value from the log-rank test. CM = the clinical model; RM-Multi = the radiomics model; DLM-Deep feature = the deep learning model; CRM-Multi = the clinical + radiomics model; CDLM-Deep feature = the clinical + deep-learning model.

Table 3 summarizes the performances of multiple risk models for BCR-free survival using fivefold cross-validation in our single-center dataset for both the training and the test sets. Figure 4 depicts the representative K–M analysis of the five models for the test sets described in Table 3. The prognostic performance of the clinical variable risk model (denoted as CM) was validated showing an HR of 5.82 (95% CI: 0.92–10.72, *p* = 0.0075), a C-index of 0.81 (95% CI, 0.69–0.92), and an iAUC of 0.85 over five folds in the test set. In comparison, the radiomics risk model based on the radiomics features extracted from mpMRI (denoted as RM-Multi) exhibited a numerically inferior predictive performance to the CM, with an HR of 2.67 (95% CI, 0.20–5.14, *p* = 0.054), C-index of 0.66 (95% CI, 0.57–0.76), and iAUC of 0.68 (Table 3 and Figure 4) in the test set.

**Figure 5:**
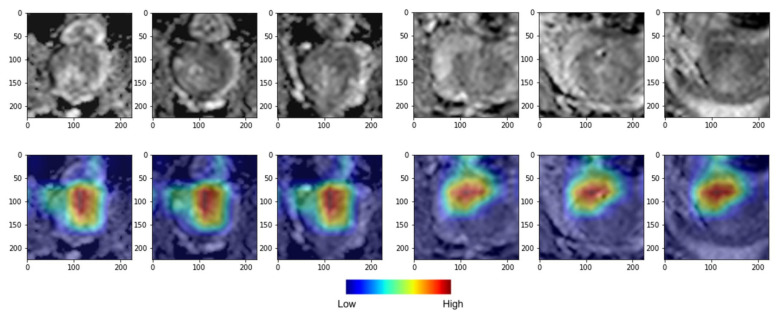
Deep learning (DL) gradient-weighted class activation mapping (Grad-CAM) examples for biochemical recurrence (BCR) positive (**left**) and BCR negative (**right**) cases. The top row shows axial slices, and the bottom row shows colored attention maps overlaid in color. Red color denotes high attention, and blue denotes low attention. Our DL model focused more on the high-attention region in red compared to the low-attention region in blue to stratify low- and high-risk groups.

Grad-CAM interpretation of our DL model confirmed its high degree of attention over regions close to the primary cancer for predicting the BCR status (Figure 5). Notably, the DL deep feature risk model (denoted as DLM-Deep feature) exhibited better predictive performance than the RM-Multi, according to the cross-validation analysis; however, it fared worse than the CM, exhibiting an HR of 4.37 (95% CI: 0.00–0.83, *p* = 0.0219), a C-index of 0.74 (95% CI 0.59–0.88), and iAUC of 0.77 in the test set, indicating that using only mpMRI imaging features without radiomics or deep features is insufficient for predicting BCR-free survival. Moreover, the combined risk model collating DL’s deep features with six clinical variables (denoted as CDLM-deep feature) yielded the best predictive performance for the 10 year BCR-free survival with an HR of 7.72 (95% CI: 1.24–14.19, *p* = 0.0008), C-index of 0.89 (95% CI: 0.77–1.00), and iAUC of 0.93 in the test set; however, the combined risk model adding six clinical variables to the RM-Multi model (denoted as CRM-Multi) did not outperform CM alone, with an HR of 5.44 (95% CI: 2.71–8.17, *p* < 0.001), C-index of 0.83 (95% CI: 0.72–0.93), and iAUC of 0.87 in the test set.

## 4. Discussion

To date, several clinical predictive tools have been proposed for post-RP BCR prediction [54,55], including preoperative tools, such as the University of California, San Francisco Cancer of the Prostate Risk Assessment (UCSF-CAPRA) [54]. However, three of the preoperative variables defining the CAPRA score (biopsy GS, clinical T stage, and percentage of positive biopsy scores) could underestimate the true GS and extent of cancer [16,56,57,58,59,60,61,62,63,64,65,66]. More recently, the CAPRA post-surgical (CAPRA-S) score, comprising PSA, surgical GS, PSM, ECE, SVI, and lymph node involvement (LNI), was introduced [55]. However, for calculating CAPRA-S, patients with no pelvic lymph node dissection (PLND) are presumed to have negative LNI [55]. Due to the underutilization of PLND in intermediate- and high-risk individuals owing to conflicting evidence on the utility of PLND [67,68], excluding those with unknown LNI status would be challenging for predicting post-RP BCR using CAPRA-S [69].

The rapid development of deep neural network algorithms has gradually improved the performance of DL, which exceeds that of radiomics for several tasks [22,23,24,25,26,27,29,30,31,32,33,34,35,36,51]. Our results confirmed the superior performance of a novel DL algorithm using deep features derived from mpMRI over conventional mpMRI-based radiomics approaches. In a retrospective study analyzing 195 patients with a high risk of PCa recurrence (median follow-up: 46.3 months), Bourbonne et al. used an MRI T2- and ADC map-derived radiomics model to predict the BCR-free survival following RP [20]. The performance of their model was lower (HR 6.8) than that of our CDLM-Deep feature model (HR: 7.7) [20]. Similarly, Li et al. adopted the radiomics approach (*n* = 198, median 35-month follow-up), which yielded a poorer prognostic performance (C-index: 0.77) than our CDLM-Deep feature model (C-index: 0.89) [70].

Our DL model included 17 layers of convolution and, thus, could effectively model a wide spectrum of features starting from fine-grained (found in early layers) to abstract (found in late layers) features compared to fixed scale features of radiomics. Moreover, our DL model included nonlinear activation functions in the intermediate layers and, thus, could model possibly nonlinear interactions among the features. Another possibility could be that the DL model adopted 320 deep features in the final layer compared to 72 radiomics features per modality, and the additional degree of freedom in the DL model might have resulted in better handling of the multimodal MRI data. Lastly, our DL model adopted the pretrained EfficientNetB0 network and, thus, required fewer samples than the standard CNN training approach. Yan et al. adopted a similar approach to ours, using DL with mpMRI features to develop a deep survival neural network for predicting BCR-free survival following RP [34]. They retrospectively enrolled 485 patients who underwent RP between 2010 and 2017 from three institutions [34]. However, the performance of their deep radiomics signature was worse (C-index: 0.80) than ours (C-index: 0.89), owing to limitations in their DL approach [34], such as not directly extracting features from raw MR images but rather using previously extracted radiomics features [34]. Similarly, Hiremath et al. constructed an integrated nomogram (ClaD) using PSA levels, prostate volume, lesion volume, prostate imaging reporting and data system score, and DL predictions from biparametric MRI to identify clinically significant PCa [35]. The ClaD nomogram resulted in a significant separation in K–M survival curves between the patients with and without BCR (HR: 5.92, *p* = 0.044) [35]; however, its performance was inferior to that of our model owing to the lack of DCE image data and its small sample size (*n* = 81).

Moreover, in principle, DL models may not require tumor segmentation if the input of the model is a full slice [71]. However, in our case, we adopted an image patch centered on the tumor centroid partly because using the patch requires less memory compared to using the full slice. Thus, we required tumor centroid information and not the full tumor segmentation information. Specifying the patch is easier to perform than providing voxel-level tumor segmentation. Hence, we reduced the expert burden and associated variability compared to using tumor segmentation in the radiomics models. We sampled three 64 × 64 image patches that enclosed the primary tumor in three axial slices to apply patch-based DL approaches focusing on the tumor ROI. Through this approach, we could easily incorporate the tumor periphery related to the TME (peritumoral region immediately surrounding the visible tumor related to the tumor-adjacent normal tissues and TME), as the DL analysis included a rectangular region comprising both the tumor ROI and the periphery [72]. Similarly, Hiremath et al. explored multiple input configurations of the DL network, by extracting patches at different scales; each subsequent scale incorporated additional information from the peritumoral region on biparametric MRI [35]. The additional information provided by out-of-lesion tissues could have improved the learning of the DL framework, since mpMRI consistently underestimates the PCa pathological tumor size/extent when extending substantially beyond the ROI surface [22,73,74]. Furthermore, peritumoral lesions, forming the TME at the intermediate and preneoplastic states, provide critical molecular information that could serve to differentiate between aggressive and indolent tumors [35,72,75,76]. Dynamic cell-to-cell interactions with the tumor, extracellular matrix, or secreted protumoral factors lead to TME heterogeneity, which plays a key role in PCa progression, distant metastasis, lethal outcomes, and therapeutic failure [75,76,77].

The EfficientNet network series demonstrated a high performance with respect to the efficiency and accuracy of ImageNet tasks [52,78]. Herein, the shortcoming of the relatively small sample size of the mpMRI input was partially mitigated by adopting the EfficientNet-B0 model [10,78]. The multimodality deep survival network based on EfficientNetB0 and clinical features demonstrated the best performance for predicting post-RP long-term BCR-free survival. We applied several optimization steps to the learned deep features to improve the prediction performance, and we combined previously known important clinical variables related to BCR to achieve complete integration of the available useful clinical information, concurrent with other studies on BCR-free survival prediction tasks [34,35,79]. Recently, a Korean Prostate Cancer Database registry-based analysis demonstrated that the PSA levels, surgical GS ≥8, adverse pathological features [PSM, SVI, ECE], and adverse laboratory features (detectable PSA levels after 6 weeks) were significant predictors of BCR-free survival and overall survival (OS) following RP in Korea [6]. On the basis of these findings, the clinicopathological characteristics, including preoperative PSA, surgical GS, PSM, ECE, and SVI, rather than the preoperative biopsy finding, were considered for constructing our prediction model. The combined model CDLM-Deep feature achieved stronger discriminatory power than any other model (CM, RM, CRM-Multi, and DLM-Deep feature) as indicated by performance indicators, such as the HR, C-index, and iAUC. The CDLM-Deep feature model performed better than the DLM-Deep feature or CM models, providing further evidence that deep features can adjust to domain-specific data and complementary information beyond clinical variables. This points to further evidence of deep features adjusting to domain-specific data and providing complementary information beyond clinical variables. Compared with the traditional predictive tools, our approach of combining DL deep features with clinical risk factors could facilitate prognostic predictions based on pre-RP mpMRI, further contributing to precision oncology and enhanced quality of care following RP in Korean patients with high-risk PCa. Additional advantages, such as a longer follow-up with larger sample sets compared to previous radiomics approaches that missed out on recent advances in DL [10,15,17,18,19,20,70,80], could improve the robustness and relevance of our study. Interestingly, the performance of the CRM-Multi model was comparable (or worse) to that of the CM model while several studies demonstrated that the clinical–radiomics combined model showed higher performance than the clinical or radiomics model alone for predicting clinically significant PCa and BCR-free survival [81,82]. Our contradictory findings suggest that radiomics features were predetermined; thus, they could not be adjusted to compensate for the possible overlap in the clinical variables. Further studies are needed to validate the improvement of predictive accuracy by incorporating additional clinical features to DL based on prostate mpMRI for post-RP long-term BCR-free prognosis.

Prostate-specific membrane antigen (PSMA) is an oncogenic transmembrane glycoprotein overexpressed on PCa cells, and higher degrees of PSMA expression are associated with higher aggressive biology associated with PCa progression and recurrence [83,84]. Quantitative PSMA positron emission tomography (PET) analysis has shown high performance in identifying clinically significant intraprostatic lesions and in providing noninvasive and objective risk stratification of patients with primary PCa [85,86,87,88,89,90,91,92,93,94,95]. Recent studies regarding radiomics combined with machine learning (ML) and DL applications in PSMA-PET demonstrate the potential feasibility of PSMA-PET as a novel tool for PCa risk stratification and characterization of its biological properties [85,86,87,88,91,93,96,97]. For example, in the prospective study by Cysouw and colleagues, ML-based analysis of pre-operative quantitative [^18^F]DCFPyL PET metrics could predict LNI and high-risk pathological tumor features in patients with intermediate- and high-risk primary PCa scheduled for robot-assisted RP with extended PLND [85]. Furthermore, combining information from mpMRI and the PSMA-PET might offer complementary information in the diagnosis of intermediate and high-risk PCa patients, as well as detect tumor recurrence, overcoming the limitation of a single technique to the analysis of the tumor and TME within the whole prostate gland [87,91,93,96,97]. For example, a recent study suggested the potential to discriminate between low- and high-risk PCa and to predict the BCR or the overall patient risk in primary PCa patients built on [^68^Ga]Ga-PSMA-11 PET/MRI radiomics and ML equally compared to preoperative invasive biopsy [91]. Therefore, the added value of ML/DL and radiomics-based PSMA-PET/mpMRI images in predicting BCR and risk stratification in patients with newly diagnosed PCa deserves to be further explored.

This study had several limitations. Firstly, single-center mpMRI imaging data performed 14–15 years ago were retrospectively evaluated. Secondly, tumor ROI delineation was manually performed by one expert. Thirdly, the lack of generalizability of the model predictions prevented adequate external and prospective validation of our prediction models. Thus, future research should focus on multicenter, prospective multifaceted studies combined with multi-omics (e.g., genomics, proteomics, and metabolomics) in large samples to assist the development of reproducible and interpretable DL risk models.

## 5. Conclusions

Collectively, the effective integration of deep features from presurgical prostate mpMRI with clinicopathological parameters was the most powerful prognostic tool for long-term post-RP BCR-free survival. Thus, our novel DL risk model could facilitate prognostication based on routine prostate mpMRI, facilitating patient stratification following RP and individualized postoperative management.

## Figures and Tables

**Table 1:** Prostate multiparametric magnetic resonance imaging acquisition details.

Parameters	T2WI	DWI	DCE MRI
Imaging planes	Axial, sagittal, and coronal	Axial	Axial
Sequence	Turbo spin-echo	Echoplanar imaging	3D fast-field echo
TR/TE (ms)	3800–4700/80–100	4400–4800/63–75	7.4/3.9
Flip angle (°)	NA	NA	25
Sense factor	2	2	1
B value (s/mm^2^)	NA	0, 1000	NA
Slice thickness (mm)	3	3	4
Slice gap (mm)	0.3–1	1	0
Matrix size	512 × 304	124 × 120	224 × 179
No. of excitations	3	4	1
FOV (mm)	200	200	200
Scan time	3 min 48 s	1 min 40 s	5 min 58 s (every 3 s with 60 repetitions)

T2WI = T2-weighted image; DWI = diffusion-weighted image; DCE = dynamic contrast-enhanced; TR = repetition time; TE = echo time; FOV = field of view; NA = not applicable.

**Table 2:** Baseline characteristics of the study participants.

Clinical Parameters	Full Cohort (*n* = 437)	Non-BCR (*n* = 327, 74.8%)	BCR (*n* = 110, 25.2%)	*p*
FU duration, months, median (IQR)	61 (24–120)	78 (45–118)	23 (9–52)	NA
10-year BCR-free survival, *n* (%)	76 (17)	75 (17)	1 (0)	
Age, years, median (IQR)	66 (62–71)	66 (62–71)	66 (61–71)	0.35
Preoperative PSA, mg/dL, mean ± SD	7.61 ± 6.45	6.35 ± 4.14	11.35 ± 9.81	<0.001
Pathologic stage, *n* (%)				
≤T2 (pT1–pT2)	312 (71)	258 (79)	54 (49)	
≥T3 (pT3–pT4)	125 (29)	69 (21)	56 (51)	
Adverse pathological features, *n* (%)				
Extracellular capsular extension	121 (28)	66 (20)	55 (50)	<0.001
Seminal vesicle invasion	27 (6)	5 (2)	22 (20)	<0.001
Positive surgical margin	78 (18)	30 (9)	48 (44)	<0.001
Pathologic GS ISUP score group, *n* (%)				<0.001
≤6 (Group 1)	92 (21)	87 (27)	5 (5)	
3 + 4 (Group 2)	232 (53)	183 (56)	49 (45)	
4 + 3 (Group 3)	75 (17)	42 (13)	33 (30)	
8 (Group 4)	13 (03)	6 (2)	5 (6)	
≥9 (Group 5)	25 (06)	9 (3)	16 (15)	
Lymph node involvement, *n* (%)				
Yes	3 (1)	0 (0)	3 (3)	0.02
No	83 (19)	48 (15)	35 (32)	<0.001
Not determined	351 (80)	279 (85)	72 (65)	<0.001

BCR = biochemical recurrence; FU = follow-up; IQR = interquartile range; SD = standard deviation; PSA = prostate-specific antigen; ECE = extracapsular extension; ISUP = International Society of Urologic Pathology; NA = not available.

**Table 3:** Head-to-head comparison between the five risk models for predicting biochemical recurrence-free survival.

CM	HR (95% CI)		*p*		C-Index (95% CI)		* iAUC	
	Train	Test	Train	Test	Train	Test	Train	Test
1-fold	4.57 [2.98, 7.00]	6.92 [2.96, 16.15]	<0.0001	<0.0001	0.81 [0.76, 0.85]	0.85 [0.77, 0.93]	0.86	0.90
2-fold	5.66 [3.69, 8.68]	4.76 [2.04, 11.11]	<0.0001	0.0007	0.82 [0.77, 0.86]	0.79 [0.67, 0.91]	0.87	0.85
3-fold	7.28 [4.74, 11.17]	2.71 [1.16, 6.31]	<0.0001	0.0365	0.84 [0.80, 0.88]	0.72 [0.60, 0.84]	0.89	0.74
4-fold	5.56 [3.63, 8.51]	5.34 [2.23, 12.77]	<0.0001	0.0004	0.82 [0.78, 0.86]	0.80 [0.70, 0.89]	0.87	0.84
5-fold	5.35 [3.49, 8.21]	9.39 [3.91, 22.55]	<0.0001	<0.0001	0.81 [0.77, 0.85]	0.87 [0.80, 0.94]	0.86	0.92
Mean ± SD	5.68 ± 0.99 [3.74, 7.62]	5.82 ± 2.50 [0.93, 10.72]	0.0000 ± 0.0000	0.0075 ± 0.0162	0.82 ± 0.01 [0.80, 0.84]	0.81 ± 0.05 [0.69, 0.92]	0.87 ± 0.01	0.85 ± 0.06
**RM-Multi**	**HR (95% CI)**		***p***		**C-Index** **(95% CI)**		* iAUC	
1-fold	1.30 [0.85, 1.97]	2.26 [0.97, 5.23]	0.2697	0.0918	0.53 [0.47, 0.60]	0.61 [0.49, 0.73]	0.57	0.66
2-fold	2.36 [1.55, 3.60]	4.68 [2.02, 10.84]	0.0001	0.0007	0.66 [0.61, 0.72]	0.73 [0.62, 0.84]	0.71	0.73
3-fold	2.32 [1.52, 3.55]	1.37 [0.59, 3.18]	0.0001	0.5985	0.67 [0.61, 0.72]	0.64 [0.52, 0.76]	0.71	0.65
4-fold	0.18 [1.53, 3.55]	2.98 [1.28, 6.91]	0.0001	0.0202	0.64 [0.58, 0.70]	0.69 [0.57, 0.80]	0.68	0.69
5-fold	0.06 [1.47, 3.40]	2.07 [0.89, 4.82]	0.0003	0.1387	0.66 [0.60, 0.72]	0.64 [0.55, 0.74]	0.69	0.67
Mean ± SD	1.24 ± 1.11 [0.00, 3.42]	2.67 ± 1.26 [0.20, 5.14]	0.0541 ± 0.1205	0.1700 ± 0.500	0.63 ± 0.06 [0.52, 0.75]	0.66 ± 0.04 [0.57, 0.76]	0.67 ± 0.06	0.68 ± 0.03
**CRM-Multi**	**HR (95% CI)**		***p***		**C-Index** **(95% CI)**		* iAUC	
1-fold	6.05 [3.93, 9.32]	6.92 [2.96, 16.15]	<0.0001	<0.0001	0.80 [0.76, 0.85]	0.85 [0.77, 0.93]	0.86	0.89
2-fold	6.66 [4.33, 10.285]	6.08 [2.62, 14.14]	<0.0001	<0.0001	0.82 [0.78, 0.86]	0.85 [0.76, 0.93]	0.87	0.88
3-fold	5.91 [3.84, 9.10]	3.28 [1.41, 7.65]	<0.0001	0.0113	0.83 [0.79, 0.87]	0.75 [0.64, 0.86]	0.89	0.78
4-fold	5.76 [3.76, 8.84]	4.97 [2.13, 11.64]	<0.0001	0.0005	0.82 [0.78, 0.86]	0.80 [0.72, 0.89]	0.87	0.84
5-fold	5.99 [3.90, 9.20]	5.95 [2.53, 13.98]	<0.0001	0.0001	0.81 [0.76, 0.85]	0.89 [0.83, 0.95]	0.86	0.93
Mean ± SD	6.07 ± 0.35 [5.40, 6.75]	5.44 ± 1.39 [2.71, 8.17]	0.0000 ± 0.0000	0.0024 ± 0.0050	0.82 ± 0.01 [0.79, 0.84]	0.83 ± 0.05 [0.72, 0.93]	0.87 ± 0.01	0.87 ± 0.05
**DLM-** **Deep Feature**	**HR (95% CI)**		***p***		**C-Index (95% CI)**		**iAUC**	
1-fold	7.67 [5.04, 11.67]	6.41 [2.72, 15.13]	<0.0001	<0.0001	0.89 [0.86, 0.92]	0.80 [0.69, 0.92]	0.91	0.83
2-fold	6.77 [3.82, 11.99]	2.52 [1.09, 5.83]	<0.0001	0.0527	0.77 [0.73, 0.82]	0.72 [0.61, 0.83]	0.84	0.74
3-fold	8.95 [5.88, 13.63]	7.27 [3.11, 16.96]	<0.0001	<0.0001	0.89 [0.86, 0.91]	0.83 [0.73, 0.93]	0.91	0.86
4-fold	4.72 [2.77, 8.04]	2.80 [1.20, 6.54]	<0.0001	0.0310	0.75 [0.70, 0.81]	0.68 [0.58, 0.79]	0.83	0.71
5-fold	3.19 [2.10, 4.86]	2.85 [1.23, 6.61]	<0.0001	0.0256	0.71 [0.65, 0.76]	0.67 [0.57, 0.78]	0.70	0.70
Mean ± SD	6.26 ± 2.31 [1.74, 10.78]	4.37 ± 2.28 [0.00, 8.83]	0.0000 ± 0.0000	0.0219 ± 0.0224	0.80 ± 0.08 [0.64, 0.97]	0.74 ± 0.06 [0.59, 0.88]	0.84 ± 0.09	0.77 ± 0.06
**CDLM-** **Deep Feature**	**HR (95% CI)**		***p***		**C-Index** **(95% CI)**		**iAUC**	
1-fold	8.43 [5.53, 12.84]	12.95 [5.45, 30.78]	<0.0001	<0.0001	0.91 [0.89, 0.94]	1.00 [1.00, 1.00]	0.94	1.00
2-fold	6.02 [3.93, 9.22]	3.78 [1.62, 8.82]	<0.0001	0.0042	0.82 [0.77, 0.86]	0.85 [0.76, 0.93]	0.87	0.90
3-fold	8.65 [5.68, 13.16]	7.65 [3.26, 17.92]	<0.0001	<0.0001	0.92 [0.90, 0.95]	0.87 [0.78, 0.96]	0.95	0.90
4-fold	6.07 [3.97, 9.28]	7.08 [2.99, 16.77]	<0.0001	<0.0001	0.82 [0.77, 0.86]	0.85 [0.77, 0.92]	0.87	0.89
5-fold	4.77 [3.13, 7.27]	7.13 [3.03, 16.76]	<0.0001	<0.0001	0.83 [0.79, 0.87]	0.89 [0.81, 0.98]	0.88	0.93
Mean ± SD	6.79 ± 1.68 [3.49, 10.09]	7.72 ± 3.30 [1.24, 14.19]	0.0000 ± 0.0000	0.0008 ± 0.0019	0.86 ± 0.05 [0.76, 0.96]	0.89 ± 0.06 [0.77, 1.00]	0.90 ± 0.04	0.93 ± 0.05

Performance measures are reported as the mean ± standard deviation (SD) from five measurements of cross-validation. * iAUC = time-dependent mean AUC; CM = the clinical model; RM-Multi = the radiomics model; DLM-Deep feature = the deep learning model; CRM-Multi = the clinical + radiomics model; CDLM-Deep feature = the clinical + deep-learning model.

## Data Availability

The data are unavailable due to privacy concerns.

## Supplementary Materials

The following supporting information can be downloaded at: https://www.mdpi.com/article/10.3390/cancers15133416/s1, Figure S1: Details of the RM-multi model in terms of selected features over five folds. Table S1: Data distribution of 5-fold cross-validation. Each fold keeps the ratio of BCR cases (around 75%).

## References

[1] Eastham J.A., Auffenberg G.B., Barocas D.A., Chou R., Crispino T., Davis J.W., Eggener S., Horwitz E.M., Kane C.J., Kirkby E. (2022). Clinically Localized Prostate Cancer: AUA/ASTRO Guideline, Part II: Principles of Active Surveillance, Principles of Surgery, and Follow-Up. J. Urol..

[2] Eastham J.A., Auffenberg G.B., Barocas D.A., Chou R., Crispino T., Davis J.W., Eggener S., Horwitz E.M., Kane C.J., Kirkby E. (2022). Clinically Localized Prostate Cancer: AUA/ASTRO Guideline, Part I: Introduction, Risk Assessment, Staging, and Risk-Based Management. J. Urol..

[3] Mottet N., van den Bergh R.C.N., Briers E., Van den Broeck T., Cumberbatch M.G., De Santis M., Fanti S., Fossati N., Gandaglia G., Gillessen S. (2021). EAU-EANM-ESTRO-ESUR-SIOG Guidelines on Prostate Cancer-2020 Update. Part 1: Screening, Diagnosis, and Local Treatment with Curative Intent. Eur. Urol..

[4] Cornford P., van den Bergh R.C.N., Briers E., Van den Broeck T., Cumberbatch M.G., De Santis M., Fanti S., Fossati N., Gandaglia G., Gillessen S. (2021). EAU-EANM-ESTRO-ESUR-SIOG Guidelines on Prostate Cancer. Part II-2020 Update: Treatment of Relapsing and Metastatic Prostate Cancer. Eur. Urol..

[5] Weiner A.B., Siebert A.L., Fenton S.E., Abida W., Agarwal N., Davis I.D., Dorff T.B., Gleave M., James N.D., Poon D.M.C. (2022). First-line Systemic Treatment of Recurrent Prostate Cancer after Primary or Salvage Local Therapy: A Systematic Review of the Literature. Eur. Urol. Oncol..

[6] Park J.S., Koo K.C., Choi I.Y., Lee J.Y., Hong J.H., Kim C.S., Lee H.M., Hong S.K., Byun S.S., Rha K.H. (2019). Stratification based on adverse laboratory/pathological features for predicting overall survival in patients undergoing radical prostatectomy: A K-CaP registry-based analysis. Medicine.

[7] Ferro M., de Cobelli O., Vartolomei M.D., Lucarelli G., Crocetto F., Barone B., Sciarra A., Del Giudice F., Muto M., Maggi M. (2021). Prostate Cancer Radiogenomics—From Imaging to Molecular Characterization. Int. J. Mol. Sci..

[8] Shaikh F., Dupont-Roettger D., Dehmeshki J., Kubassova O., Quraishi M.I. (2020). Advanced Imaging of Biochemical Recurrent Prostate Cancer with PET, MRI, and Radiomics. Front. Oncol..

[9] Santoro A.A., Di Gianfrancesco L., Racioppi M., Pinto F., Palermo G., Sacco E., Campetella M., Scarciglia E., Bientinesi R., Di Paola V. (2021). Multiparametric magnetic resonance imaging of the prostate: Lights and shadows. Urologia.

[10] Chaddad A., Kucharczyk M.J., Cheddad A., Clarke S.E., Hassan L., Ding S., Rathore S., Zhang M., Katib Y., Bahoric B. (2021). Magnetic Resonance Imaging Based Radiomic Models of Prostate Cancer: A Narrative Review. Cancers.

[11] Iacob R., Stoicescu E.R., Cerbu S., Manolescu D.L., Bardan R., Cumpanas A. (2023). Could Biparametric MRI Replace Multiparametric MRI in the Management of Prostate Cancer?. Life.

[12] Nematollahi H., Moslehi M., Aminolroayaei F., Maleki M., Shahbazi-Gahrouei D. (2023). Diagnostic Performance Evaluation of Multiparametric Magnetic Resonance Imaging in the Detection of Prostate Cancer with Supervised Machine Learning Methods. Diagnostics.

[13] Rajwa P., Mori K., Huebner N.A., Martin D.T., Sprenkle P.C., Weinreb J.C., Ploussard G., Pradere B., Shariat S.F., Leapman M.S. (2021). The Prognostic Association of Prostate MRI PI-RADS v2 Assessment Category and Risk of Biochemical Recurrence after Definitive Local Therapy for Prostate Cancer: A Systematic Review and Meta-Analysis. J. Urol..

[14] Telecan T., Andras I., Crisan N., Giurgiu L., Cata E.D., Caraiani C., Lebovici A., Boca B., Balint Z., Diosan L. (2022). More than Meets the Eye: Using Textural Analysis and Artificial Intelligence as Decision Support Tools in Prostate Cancer Diagnosis-A Systematic Review. J. Pers. Med..

[15] Midiri F., Vernuccio F., Purpura P., Alongi P., Bartolotta T.V. (2021). Multiparametric MRI and Radiomics in Prostate Cancer: A Review of the Current Literature. Diagnostics.

[16] Ferro M., de Cobelli O., Musi G., Del Giudice F., Carrieri G., Busetto G.M., Falagario U.G., Sciarra A., Maggi M., Crocetto F. (2022). Radiomics in prostate cancer: An up-to-date review. Ther. Adv. Urol..

[17] Spohn S.K.B., Bettermann A.S., Bamberg F., Benndorf M., Mix M., Nicolay N.H., Fechter T., Holscher T., Grosu R., Chiti A. (2021). Radiomics in prostate cancer imaging for a personalized treatment approach—Current aspects of methodology and a systematic review on validated studies. Theranostics.

[18] Cho H.H., Kim C.K., Park H. (2022). Overview of radiomics in prostate imaging and future directions. Br. J. Radiol..

[19] Cutaia G., La Tona G., Comelli A., Vernuccio F., Agnello F., Gagliardo C., Salvaggio L., Quartuccio N., Sturiale L., Stefano A. (2021). Radiomics and Prostate MRI: Current Role and Future Applications. J. Imaging.

[20] Bourbonne V., Fournier G., Vallieres M., Lucia F., Doucet L., Tissot V., Cuvelier G., Hue S., Le Penn Du H., Perdriel L. (2020). External Validation of an MRI-Derived Radiomics Model to Predict Biochemical Recurrence after Surgery for High-Risk Prostate Cancer. Cancers.

[21] Kendrick J., Francis R., Hassan G.M., Rowshanfarzad P., Jeraj R., Kasisi C., Rusanov B., Ebert M. (2021). Radiomics for Identification and Prediction in Metastatic Prostate Cancer: A Review of Studies. Front. Oncol..

[22] Bertelli E., Mercatelli L., Marzi C., Pachetti E., Baccini M., Barucci A., Colantonio S., Gherardini L., Lattavo L., Pascali M.A. (2021). Machine and Deep Learning Prediction of Prostate Cancer Aggressiveness Using Multiparametric MRI. Front. Oncol..

[23] Liberini V., Laudicella R., Balma M., Nicolotti D.G., Buschiazzo A., Grimaldi S., Lorenzon L., Bianchi A., Peano S., Bartolotta T.V. (2022). Radiomics and artificial intelligence in prostate cancer: New tools for molecular hybrid imaging and theragnostics. Eur. Radiol. Exp..

[24] Michaely H.J., Aringhieri G., Cioni D., Neri E. (2022). Current Value of Biparametric Prostate MRI with Machine-Learning or Deep-Learning in the Detection, Grading, and Characterization of Prostate Cancer: A Systematic Review. Diagnostics.

[25] Belue M.J., Turkbey B. (2022). Tasks for artificial intelligence in prostate MRI. Eur. Radiol. Exp..

[26] Twilt J.J., van Leeuwen K.G., Huisman H.J., Futterer J.J., de Rooij M. (2021). Artificial Intelligence Based Algorithms for Prostate Cancer Classification and Detection on Magnetic Resonance Imaging: A Narrative Review. Diagnostics.

[27] Naik N., Tokas T., Shetty D.K., Hameed B.M.Z., Shastri S., Shah M.J., Ibrahim S., Rai B.P., Chlosta P., Somani B.K. (2022). Role of Deep Learning in Prostate Cancer Management: Past, Present and Future Based on a Comprehensive Literature Review. J. Clin. Med..

[28] Alhasan A.S. (2021). Clinical Applications of Artificial Intelligence, Machine Learning, and Deep Learning in the Imaging of Gliomas: A Systematic Review. Cureus.

[29] Erickson B.J. (2021). Basic Artificial Intelligence Techniques: Machine Learning and Deep Learning. Radiol. Clin..

[30] Checcucci E., Autorino R., Cacciamani G.E., Amparore D., De Cillis S., Piana A., Piazzolla P., Vezzetti E., Fiori C., Veneziano D. (2020). Artificial intelligence and neural networks in urology: Current clinical applications. Minerva Urol. Nefrol..

[31] Montagnon E., Cerny M., Cadrin-Chenevert A., Hamilton V., Derennes T., Ilinca A., Vandenbroucke-Menu F., Turcotte S., Kadoury S., Tang A. (2020). Deep learning workflow in radiology: A primer. Insights Imaging.

[32] Iglesias L.L., Bellon P.S., Del Barrio A.P., Fernandez-Miranda P.M., Gonzalez D.R., Vega J.A., Mandly A.A.G., Blanco J.A.P. (2021). A primer on deep learning and convolutional neural networks for clinicians. Insights Imaging.

[33] Shah U., Biswas M.R., Alzubaidi M.S., Ali H., Alam T., Househ M., Shah Z. (2022). Recent Developments in Artificial Intelligence-Based Techniques for Prostate Cancer Detection: A Scoping Review. Stud. Health Technol. Inform..

[34] Yan Y., Shao L., Liu Z., He W., Yang G., Liu J., Xia H., Zhang Y., Chen H., Liu C. (2021). Deep Learning with Quantitative Features of Magnetic Resonance Images to Predict Biochemical Recurrence of Radical Prostatectomy: A Multi-Center Study. Cancers.

[35] Hiremath A., Shiradkar R., Fu P., Mahran A., Rastinehad A.R., Tewari A., Tirumani S.H., Purysko A., Ponsky L., Madabhushi A. (2021). An integrated nomogram combining deep learning, Prostate Imaging-Reporting and Data System (PI-RADS) scoring, and clinical variables for identification of clinically significant prostate cancer on biparametric MRI: A retrospective multicentre study. Lancet Digit. Health.

[36] Corradini D., Brizi L., Gaudiano C., Bianchi L., Marcelli E., Golfieri R., Schiavina R., Testa C., Remondini D. (2021). Challenges in the Use of Artificial Intelligence for Prostate Cancer Diagnosis from Multiparametric Imaging Data. Cancers.

[37] Simmons M.N., Stephenson A.J., Klein E.A. (2007). Natural history of biochemical recurrence after radical prostatectomy: Risk assessment for secondary therapy. Eur. Urol..

[38] Sakellakis M., Jacqueline Flores L., Ramachandran S. (2022). Patterns of indolence in prostate cancer (Review). Exp. Ther. Med..

[39] Kang D.I., Chung J.I., Ha H.K., Min K., Yoon J., Kim W., Seo W.I., Kang P., Jung S.J., Kim I.Y. (2013). Korean prostate cancer patients have worse disease characteristics than their American counterparts. Asian Pac. J. Cancer Prev..

[40] Jeong I.G., Dajani D., Verghese M., Hwang J., Cho Y.M., Hong J.H., Kim C.S., Ahn H., Ro J.Y. (2016). Differences in the aggressiveness of prostate cancer among Korean, Caucasian, and African American men: A retrospective cohort study of radical prostatectomy. Urol. Oncol..

[41] Ahn H., Kim H.J., Jeon S.S., Kwak C., Sung G.T., Kwon T.G., Park J.Y., Paick S.H. (2017). Establishment of Korean prostate cancer database by the Korean Urological Oncology Society. Investig. Clin. Urol..

[42] Tanaka N., Nakai Y., Miyake M., Anai S., Inoue T., Fujii T., Konishi N., Fujimoto K. (2017). Trends in risk classification and primary therapy of Japanese patients with prostate cancer in Nara urological research and treatment group (NURTG)—Comparison between 2004–2006, 2007–2009, and 2010–2012. BMC Cancer.

[43] Seo W.I., Kang P.M., Kang D.I., Yoon J.H., Kim W., Chung J.I. (2014). Cancer of the Prostate Risk Assessment (CAPRA) Preoperative Score Versus Postoperative Score (CAPRA-S): Ability to predict cancer progression and decision-making regarding adjuvant therapy after radical prostatectomy. J. Korean Med. Sci..

[44] Hu M.B., Yang T., Hu J.M., Zhu W.H., Jiang H.W., Ding Q. (2018). Prognostic factors in Chinese patients with prostate cancer receiving primary androgen deprivation therapy: Validation of Japan Cancer of the Prostate Risk Assessment (J-CAPRA) score and impacts of pre-existing obesity and diabetes mellitus. Int. J. Clin. Oncol..

[45] Tilki D., Mandel P., Schlomm T., Chun F.K., Tennstedt P., Pehrke D., Haese A., Huland H., Graefen M., Salomon G. (2015). External validation of the CAPRA-S score to predict biochemical recurrence, metastasis and mortality after radical prostatectomy in a European cohort. J. Urol..

[46] Park J.J., Kim C.K., Park S.Y., Park B.K., Lee H.M., Cho S.W. (2014). Prostate cancer: Role of pretreatment multiparametric 3-T MRI in predicting biochemical recurrence after radical prostatectomy. AJR Am. J. Roentgenol..

[47] Cookson M.S., Aus G., Burnett A.L., Canby-Hagino E.D., D’Amico A.V., Dmochowski R.R., Eton D.T., Forman J.D., Goldenberg S.L., Hernandez J. (2007). Variation in the definition of biochemical recurrence in patients treated for localized prostate cancer: The American Urological Association Prostate Guidelines for Localized Prostate Cancer Update Panel report and recommendations for a standard in the reporting of surgical outcomes. J. Urol..

[48] Bang S., Yu J., Chung J.H., Song W., Kang M., Sung H.H., Jeon H.G., Jeong B.C., Seo S.I., Lee H.M. (2021). Usefulness of MRI targeted prostate biopsy for detecting clinically significant prostate cancer in men with low prostate-specific antigen levels. Sci. Rep..

[49] Epstein J.I., Egevad L., Amin M.B., Delahunt B., Srigley J.R., Humphrey P.A., Grading C. (2016). The 2014 International Society of Urological Pathology (ISUP) Consensus Conference on Gleason Grading of Prostatic Carcinoma: Definition of Grading Patterns and Proposal for a New Grading System. Am. J. Surg. Pathol..

[50] Van Griethuysen J.J.M., Fedorov A., Parmar C., Hosny A., Aucoin N., Narayan V., Beets-Tan R.G.H., Fillion-Robin J.C., Pieper S., Aerts H. (2017). Computational Radiomics System to Decode the Radiographic Phenotype. Cancer Res..

[51] Cheng P.M., Montagnon E., Yamashita R., Pan I., Cadrin-Chenevert A., Perdigon Romero F., Chartrand G., Kadoury S., Tang A. (2021). Deep Learning: An Update for Radiologists. Radiographics.

[52] Wang J., Liu Q., Xie H., Yang Z., Zhou H. (2021). Boosted EfficientNet: Detection of Lymph Node Metastases in Breast Cancer Using Convolutional Neural Networks. Cancers.

[53] Tan M., Le Q.V. EfficientNet: Rethinking Model Scaling for Convolutional Neural Networks. Proceedings of the 36th International Conference on Machine Learning (ICML).

[54] Cooperberg M.R., Pasta D.J., Elkin E.P., Litwin M.S., Latini D.M., Du Chane J., Carroll P.R. (2005). The University of California, San Francisco Cancer of the Prostate Risk Assessment score: A straightforward and reliable preoperative predictor of disease recurrence after radical prostatectomy. J. Urol..

[55] Cooperberg M.R., Hilton J.F., Carroll P.R. (2011). The CAPRA-S score: A straightforward tool for improved prediction of outcomes after radical prostatectomy. Cancer.

[56] Cohen M.S., Hanley R.S., Kurteva T., Ruthazer R., Silverman M.L., Sorcini A., Hamawy K., Roth R.A., Tuerk I., Libertino J.A. (2008). Comparing the Gleason prostate biopsy and Gleason prostatectomy grading system: The Lahey Clinic Medical Center experience and an international meta-analysis. Eur. Urol..

[57] Kuroiwa K., Shiraishi T., Naito S., Clinicopathological Research Group for Localized Prostate Cancer (2011). Gleason score correlation between biopsy and prostatectomy specimens and prediction of high-grade Gleason patterns: Significance of central pathologic review. Urology.

[58] Epstein J.I., Feng Z., Trock B.J., Pierorazio P.M. (2012). Upgrading and downgrading of prostate cancer from biopsy to radical prostatectomy: Incidence and predictive factors using the modified Gleason grading system and factoring in tertiary grades. Eur. Urol..

[59] Sfoungaristos S., Perimenis P. (2013). Clinical and pathological variables that predict changes in tumour grade after radical prostatectomy in patients with prostate cancer. Can. Urol. Assoc. J..

[60] Scattoni V., Maccagnano C., Capitanio U., Gallina A., Briganti A., Montorsi F. (2014). Random biopsy: When, how many and where to take the cores?. World J. Urol..

[61] Van Praet C., Libbrecht L., D’Hondt F., Decaestecker K., Fonteyne V., Verschuere S., Rottey S., Praet M., De Visschere P., Lumen N. (2014). Agreement of Gleason score on prostate biopsy and radical prostatectomy specimen: Is there improvement with increased number of biopsy cylinders and the 2005 revised Gleason scoring?. Clin. Genitourin. Cancer.

[62] Schreiber D., Wong A.T., Rineer J., Weedon J., Schwartz D. (2015). Prostate biopsy concordance in a large population-based sample: A Surveillance, Epidemiology and End Results study. J. Clin. Pathol..

[63] Beckmann K., O’Callaghan M., Vincent A., Cohen P., Borg M., Roder D., Evans S., Millar J., Moretti K. (2019). Extent and predictors of grade upgrading and downgrading in an Australian cohort according to the new prostate cancer grade groupings. Asian J. Urol..

[64] Calio B.P., Sidana A., Sugano D., Gaur S., Maruf M., Jain A.L., Merino M.J., Choyke P.L., Wood B.J., Pinto P.A. (2018). Risk of Upgrading from Prostate Biopsy to Radical Prostatectomy Pathology-Does Saturation Biopsy of Index Lesion during Multiparametric Magnetic Resonance Imaging-Transrectal Ultrasound Fusion Biopsy Help?. J. Urol..

[65] Dolatkhah S., Mirtalebi M., Daneshpajouhnejad P., Barahimi A., Mazdak H., Izadpanahi M.H., Mohammadi M., Taheri D. (2019). Discrepancies between Biopsy Gleason Score and Radical Prostatectomy Specimen Gleason Score: An Iranian Experience. Urol. J..

[66] Jang W.S., Koh D.H., Kim J., Lee J.S., Chung D.Y., Ham W.S., Rha K.H., Choi Y.D. (2019). The prognostic impact of downgrading and upgrading from biopsy to radical prostatectomy among men with Gleason score 7 prostate cancer. Prostate.

[67] Malkiewicz B., Kielb P., Karwacki J., Czerwinska R., Dlugosz P., Leminski A., Nowak L., Krajewski W., Szydelko T. (2022). Utility of Lymphadenectomy in Prostate Cancer: Where Do We Stand?. J. Clin. Med..

[68] Cheung D.C., Fleshner N., Sengupta S., Woon D. (2020). A narrative review of pelvic lymph node dissection in prostate cancer. Transl. Androl. Urol..

[69] Lorent M., Maalmi H., Tessier P., Supiot S., Dantan E., Foucher Y. (2019). Meta-analysis of predictive models to assess the clinical validity and utility for patient-centered medical decision making: Application to the CAncer of the Prostate Risk Assessment (CAPRA). BMC Med. Inform. Decis. Mak..

[70] Li L., Shiradkar R., Leo P., Algohary A., Fu P., Tirumani S.H., Mahran A., Buzzy C., Obmann V.C., Mansoori B. (2021). A novel imaging based Nomogram for predicting post-surgical biochemical recurrence and adverse pathology of prostate cancer from pre-operative bi-parametric MRI. EBioMedicine.

[71] Lee S.H., Cho H.H., Lee H.Y., Park H. (2019). Clinical impact of variability on CT radiomics and suggestions for suitable feature selection: A focus on lung cancer. Cancer Imaging.

[72] Yoon H.J., Kang J., Park H., Sohn I., Lee S.H., Lee H.Y. (2020). Deciphering the tumor microenvironment through radiomics in non-small cell lung cancer: Correlation with immune profiles. PLoS ONE.

[73] Priester A., Natarajan S., Khoshnoodi P., Margolis D.J., Raman S.S., Reiter R.E., Huang J., Grundfest W., Marks L.S. (2017). Magnetic Resonance Imaging Underestimation of Prostate Cancer Geometry: Use of Patient Specific Molds to Correlate Images with Whole Mount Pathology. J. Urol..

[74] Pooli A., Johnson D.C., Shirk J., Markovic D., Sadun T.Y., Sisk A.E., Mohammadian Bajgiran A., Afshari Mirak S., Felker E.R., Hughes A.K. (2021). Predicting Pathological Tumor Size in Prostate Cancer Based on Multiparametric Prostate Magnetic Resonance Imaging and Preoperative Findings. J. Urol..

[75] Zhou R., Feng Y., Ye J., Han Z., Liang Y., Chen Q., Xu X., Huang Y., Jia Z., Zhong W. (2021). Prediction of Biochemical Recurrence-Free Survival of Prostate Cancer Patients Leveraging Multiple Gene Expression Profiles in Tumor Microenvironment. Front. Oncol..

[76] Gevaert T., Van Eycke Y.R., Vanden Broeck T., Van Poppel H., Salmon I., Rorive S., Muilwijk T., Claessens F., De Ridder D., Joniau S. (2020). The potential of tumour microenvironment markers to stratify the risk of recurrence in prostate cancer patients. PLoS ONE.

[77] Ge R., Wang Z., Cheng L. (2022). Tumor microenvironment heterogeneity an important mediator of prostate cancer progression and therapeutic resistance. NPJ Precis. Oncol..

[78] Stollmayer R., Budai B.K., Ronaszeki A., Zsombor Z., Kalina I., Hartmann E., Toth G., Szoldan P., Berczi V., Maurovich-Horvat P. (2022). Focal Liver Lesion MRI Feature Identification Using Efficientnet and MONAI: A Feasibility Study. Cells.

[79] Li H., Lee C.H., Chia D., Lin Z., Huang W., Tan C.H. (2022). Machine Learning in Prostate MRI for Prostate Cancer: Current Status and Future Opportunities. Diagnostics.

[80] Shiradkar R., Ghose S., Mahran A., Li L., Hubbard I., Fu P., Tirumani S.H., Ponsky L., Purysko A., Madabhushi A. (2022). Prostate Surface Distension and Tumor Texture Descriptors from Pre-Treatment MRI Are Associated with Biochemical Recurrence Following Radical Prostatectomy: Preliminary Findings. Front. Oncol..

[81] Shiradkar R., Ghose S., Jambor I., Taimen P., Ettala O., Purysko A.S., Madabhushi A. (2018). Radiomic features from pretreatment biparametric MRI predict prostate cancer biochemical recurrence: Preliminary findings. J. Magn. Reson. Imaging.

[82] Bourbonne V., Vallieres M., Lucia F., Doucet L., Visvikis D., Tissot V., Pradier O., Hatt M., Schick U. (2019). MRI-Derived Radiomics to Guide Post-operative Management for High-Risk Prostate Cancer. Front. Oncol..

[83] Israeli R.S., Powell C.T., Corr J.G., Fair W.R., Heston W.D. (1994). Expression of the prostate-specific membrane antigen. Cancer Res..

[84] Kaittanis C., Andreou C., Hieronymus H., Mao N., Foss C.A., Eiber M., Weirich G., Panchal P., Gopalan A., Zurita J. (2018). Prostate-specific membrane antigen cleavage of vitamin B9 stimulates oncogenic signaling through metabotropic glutamate receptors. J. Exp. Med..

[85] Cysouw M.C.F., Jansen B.H.E., van de Brug T., Oprea-Lager D.E., Pfaehler E., de Vries B.M., van Moorselaar R.J.A., Hoekstra O.S., Vis A.N., Boellaard R. (2021). Machine learning-based analysis of [(18)F]DCFPyL PET radiomics for risk stratification in primary prostate cancer. Eur. J. Nucl. Med. Mol. Imaging.

[86] Leung K.H., Rowe S.P., Leal J.P., Ashrafinia S., Sadaghiani M.S., Chung H.W., Dalaie P., Tulbah R., Yin Y., VanDenBerg R. (2022). Deep learning and radiomics framework for PSMA-RADS classification of prostate cancer on PSMA PET. EJNMMI Res..

[87] Chan T.H., Haworth A., Wang A., Osanlouy M., Williams S., Mitchell C., Hofman M.S., Hicks R.J., Murphy D.G., Reynolds H.M. (2023). Detecting localised prostate cancer using radiomic features in PSMA PET and multiparametric MRI for biologically targeted radiation therapy. EJNMMI Res..

[88] Pozaruk A., Pawar K., Li S., Carey A., Cheng J., Sudarshan V.P., Cholewa M., Grummet J., Chen Z., Egan G. (2021). Augmented deep learning model for improved quantitative accuracy of MR-based PET attenuation correction in PSMA PET-MRI prostate imaging. Eur. J. Nucl. Med. Mol. Imaging.

[89] Zamboglou C., Carles M., Fechter T., Kiefer S., Reichel K., Fassbender T.F., Bronsert P., Koeber G., Schilling O., Ruf J. (2019). Radiomic features from PSMA PET for non-invasive intraprostatic tumor discrimination and characterization in patients with intermediate- and high-risk prostate cancer—A comparison study with histology reference. Theranostics.

[90] Liu C., Liu T., Zhang N., Liu Y., Li N., Du P., Yang Y., Liu M., Gong K., Yang X. (2018). (68)Ga-PSMA-617 PET/CT: A promising new technique for predicting risk stratification and metastatic risk of prostate cancer patients. Eur. J. Nucl. Med. Mol. Imaging.

[91] Papp L., Spielvogel C.P., Grubmuller B., Grahovac M., Krajnc D., Ecsedi B., Sareshgi R.A.M., Mohamad D., Hamboeck M., Rausch I. (2021). Supervised machine learning enables non-invasive lesion characterization in primary prostate cancer with [(68)Ga]Ga-PSMA-11 PET/MRI. Eur. J. Nucl. Med. Mol. Imaging.

[92] Feliciani G., Celli M., Ferroni F., Menghi E., Azzali I., Caroli P., Matteucci F., Barone D., Paganelli G., Sarnelli A. (2022). Radiomics Analysis on [(68)Ga]Ga-PSMA-11 PET and MRI-ADC for the Prediction of Prostate Cancer ISUP Grades: Preliminary Results of the BIOPSTAGE Trial. Cancers.

[93] Meyer A.R., Joice G.A., Allaf M.E., Rowe S.P., Gorin M.A. (2018). Integration of PSMA-targeted PET imaging into the armamentarium for detecting clinically significant prostate cancer. Curr. Opin. Urol..

[94] Bouchelouche K., Choyke P.L. (2018). Advances in prostate-specific membrane antigen PET of prostate cancer. Curr. Opin. Oncol..

[95] Hofman M.S., Iravani A., Nzenza T., Murphy D.G. (2018). Advances in Urologic Imaging: Prostate-Specific Membrane Antigen Ligand PET Imaging. Urol. Clin..

[96] Emmett L., Buteau J., Papa N., Moon D., Thompson J., Roberts M.J., Rasiah K., Pattison D.A., Yaxley J., Thomas P. (2021). The Additive Diagnostic Value of Prostate-specific Membrane Antigen Positron Emission Tomography Computed Tomography to Multiparametric Magnetic Resonance Imaging Triage in the Diagnosis of Prostate Cancer (PRIMARY): A Prospective Multicentre Study. Eur. Urol..

[97] Guglielmo P., Marturano F., Bettinelli A., Gregianin M., Paiusco M., Evangelista L. (2021). Additional Value of PET Radiomic Features for the Initial Staging of Prostate Cancer: A Systematic Review from the Literature. Cancers.

