# Supplementary material for: Novel Multiparametric Magnetic Resonance Imaging-Based Deep Learning and Clinical Parameter Integration for the Prediction of Long-Term Biochemical Recurrence-Free Survival in Prostate Cancer after Radical Prostatectomy
Source: Cancers (Basel). 2023 Jun 29;15(13):3416. doi: 10.3390/cancers15133416 (PMC10340407; doi:10.3390/cancers15133416)
Supplement: Supplementary file 1 [file cancers-15-03416-s001.zip › cancers-2420794-supplementary.pdf]

Supplementary Materials

Table S1. Data distribution of 5-fold cross-validation. Each fold keeps the ratio of BCR cases (around 75%).

Data Split	Patients	1-fold	2-fold	3-fold	4-fold	5-fold
**Train Data**	Total	350	349	349	350	350
	BCR	88	88	88	88	88
	No BCR	262	261	261	262	262
**Test Data**	Total	87	88	88	87	87
	BCR	22	22	22	22	22
	No BCR	65	66	66	65	65

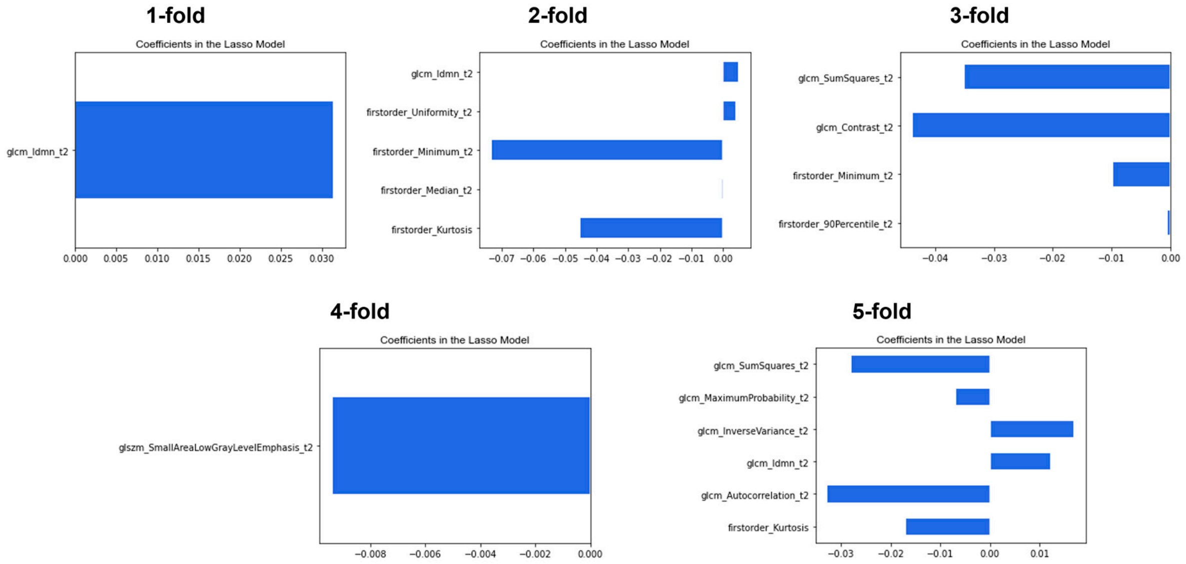

Figure S1. Details of the RM-multi model in terms of selected features over five folds.
